# The prevalence and risk factors of urinary incontinence amongst Palestinian women with type 2 diabetes mellitus: A cross-sectional study

**DOI:** 10.1080/2090598X.2019.1699340

**Published:** 2019-12-09

**Authors:** Zaher Nazzal, Batool Khatib, Bayan Al-Quqa, Lina Abu-Taha, Ahmad Jaradat

**Affiliations:** a Department of Family and Community Medicine, Faculty of Medicine and Health Sciences, An-Najah National University, Nablus, Palestine; b Faculty of Medicine and Health Sciences, An-Najah National University, Nablus, Palestine; c Department of Urology, An-Najah National University Hospital, Nablus, Palestine

**Keywords:** Urinary incontinence, prevalence, female, type 2 diabetes mellitus, Palestine

## Abstract

**Objective**: To determine the prevalence of urinary incontinence (UI) in women with type 2 diabetes mellitus (T2DM) in the North West Bank, Palestine, and to assess the role of potential risk factors including age and DM control.

**Patients and methods**: Adult women with DM attending governmental primary healthcare centres in the North West Bank were interviewed using the Centers for Disease Control and Prevention National Health and Nutrition Examination Survey (NHANES) standardised UI questionnaire. The prevalence of UI was estimated and differences between groups were evaluated using the chi-square test. A multivariate logistic model was used to estimate the adjusted relationships and to control for confounders. The statistical significance level was set at *P* < 0.05. The study was approved by the Institutional Review Board at An-Najah National University.

**Results**: The study included 381 women with T2DM, aged 30–83 years, of whom 43.2% (95% confidence interval [CI] = 37.9–47.8%) reported UI regardless of the type. About 40% reported that they were extremely bothered by the condition and 35.2% stated that their daily routine life was greatly affected. Amongst the women with UI, 133 (80.6%) and 128 (77.6%) were found to have urge and stress UI, respectively. UI was found to be significantly associated with a history of recurrent urinary tract infection (adjusted odds ratio [OR] 3.0, 95% CI 1.9–4.9; *P* < 0.001) and parity (adjusted OR 1.7, 95% CI 1.1–2.7; *P* = 0.04)

**Conclusions**: The prevalence of UI amongst Palestinian women with T2DM regardless of the type is high. The findings highlight the importance of educating women with T2DM about UI. The medical team should focus on this problem as it is often neglected; physicians should be alert for UI as it is often underreported and therefore undertreated.

**Abbreviations:** BMI: body mass index; (T2)DM: (type 2) diabetes mellitus; HbA_1c_: haemoglobin A_1c_; MoH: Ministry of Health; NHANES: National Health and Nutrition Examination Survey; OR: odds ratio; QoL: quality of life; (S)(U)UI: (stress) (urge) urinary incontinence.

## Introduction

Diabetes mellitus (DM) is one of the most common chronic medical illnesses worldwide, with rising incidence and prevalence [1]. DM can lead to important microvascular and macrovascular complications; such as cardiovascular diseases, neuropathy, retinopathy, and nephropathy. Recent estimates show that by 2025 women with type 2 DM (T2DM) will represent the highest percentage of the diabetic population [2].

Urinary incontinence (UI) remains a highly prevalent cross‐cultural and costly condition that affects women of all ages. In the Middle East, 20–60% of women complain of UI [3,4]. The ICS defines UI as ‘the complaint of any involuntary leakage of urine’ [5]. It was predicted that 423 million individuals would suffer from UI by the year 2018 worldwide.

Although UI is not a life-threatening condition, it affects the general well-being of women, especially emotional and social aspects. It decreases quality of life (QoL), and has been associated with many poor outcomes [6]. The urge and frequent toilet visits may lead to higher incidence of falls causing fractures, especially hip fracture in elderly women and its associated complications. In addition, UI can lead to recurrent or persistent UTI [7] that could cause sepsis and septic shock. Moreover, older people with UI consume more healthcare resources than those without, consequently presenting a substantial burden on healthcare systems. Additionally, UI has been associated with depression, decreased work productivity, and a decline in QoL [8].

DM has been shown to be an independent risk factor for UI. In fact, it may be an earlier and more common consequence of hyperglycaemia than other microvascular complications such as retinopathy, neuropathy, or nephropathy [9]. A likely cause of UI is microvascular damage to the innervations of the bladder and urethral sphincter, sphincter dysfunction, bladder instability, urinary retention, and elevated postvoid residual urine volume that contribute to overflow UI, chronic bacterial colonisation, and UTIs [10].

Many different patient background and clinical characteristics have been identified as risk factors for UI. Age is a significant risk factor for UI, as ageing is associated with decreased sensation, detrusor muscle mass, elasticity, and capacity of the bladder [11]. Obesity, as well, increases the risk of UI amongst diabetic women because it leads to increased intra-abdominal and pelvic pressure [11]. The presence of frequent UTIs and the number of normal vaginal deliveries (parity) are other important risk factors for UI [7]. Patients’ clinical variables have also been identified in epidemiological studies as important risk factors for UI and include the duration of DM, haemoglobin A_1c_ (HbA_1c_) level, and presence of diabetic long-term complications [12].

In Palestine, the prevalence of T2DM is increasing tremendously. It affected 15.3% of the population in 2010, and this is predicted to increase to 20.8% of the general population in 2020 and to 23.4% by 2030 [13]. No previous studies were found on the burden of UI amongst Palestinian women and its risk factors. The present study aimed to determine the prevalence of UI amongst women with T2DM and to find the risk factors that put diabetic women at risk of developing UI. Furthermore, the present study was undertaken to explore the impact of UI on the lives of Palestinian diabetic women, which may help in designing appropriate intervention strategies to optimise health and well-being for this population.

## Patients and methods

A quantitative cross-sectional study design was used to assess and determine the prevalence of UI amongst Palestinian women with T2DM. Almost all Palestinian patients with DM are followed in the diabetic clinics at Primary Health Centres of the Ministry of Health (MoH), where they are offered all services related to prevention, management, and control of DM. One governmental diabetic clinic, located in the primary health directorate, is available in each district, where all patients with DM in that district are referred to. The study was conducted in six Primary Health Centres in all cities of the North West Bank (Jenin, Tubas, Tulkarem, Nablus, Qalqilya, and Salfit). Patients with type 1 DM, male patients, and those aged <18 years were excluded.

### Data collection

The sample size formula for prevalence studies was used to calculate the sample size, in which statistics for a 95% level of confidence is 1.96, the expected proportion (in s proportion of one) is 0.5, and the precision (in s proportion of one) is 0.05. The sample size was 381 diabetic women selected through a systemic sampling technique, taking into consideration patients who met inclusion and exclusion criteria upon registration in their follow-up clinics.

An interviewer-administered questionnaire, adapted from The National Health and Nutrition Examination Survey (NHANES), was used to obtain information on UI. It is a standardised survey that has been used in previous studies to assess UI and its effect on a patient’s life. It was chosen because it is easy to administer, validated in several studies, and found to be reliable for UI diagnosis. The Arabic version of the questionnaire was pretested and validated in Jordan by Bani-Isa et al. [3], which has a similar culture as Palestine.

The questionnaire was reviewed by three urologists to ensure its face and content validity. Additionally, it was pre-tested with a group of patients and ambiguous questions were rephrased for clarity. It consists of two parts; part one includes questions on background information, daily life-style, medical history, and T2DM status. Age was categorised as <50, 50–59.9, 60–69.9, and >70 years; parity as <5 and ≥5 children; and body mass index (BMI) as ˂30 and ≥30 kg/m^2^. Physical activity was defined according to the WHO recommendation as 150 min/week. DM-related variables were collected: HbA_1c_ level <7 and ≥7%; duration of T2DM <10 and ≥10 years; and type of treatment, insulin and non-insulin.

The second part covers signs and symptoms of UI, frequency, and severity. UI is defined, by The AUA, as any leak or loss of control of urine even of small amounts. Women were asked on the questionnaires, ‘During the last 12 months, how often have you leaked or lost control of your urine?’; response categories were ‘never’, ‘<1 time/month’, ‘2–3 times/month’, ‘1 time/week’, and ‘1 time/day’. Of women who reported leaking, we asked, ‘When you lose your urine, how much usually leaks?’; women could indicate a ‘few drops’, ‘enough to wet your underwear’, ‘enough to wet your outer clothing’ and ‘enough to wet the ﬂoor’. UI cases were classiﬁed as any UI if women reported leaking ≥1 time/month and as frequent UI if they reported leaking ≥1 time/week. Women’s responses to these questions have been shown to be highly reliable. We also collected more information on UI type amongst the women. Stress UI (SUI) was defined as UI occurring with an activity, i.e. coughing, lifting, or exercise. While urge UI (UUI) was defined as UI occurring with an urge or pressure to urinate and not getting to a toilet fast enough [10].

Approval of the study was obtained from the Institutional Review Board at An-Najah National University. Additionally, official permission from the Palestinian MoH was obtained to conduct the study at Primary Health Centres. Patients were invited to participate in the study voluntarily after the nature and purpose of the study were explained to them. The interview took place in a closed clinic in order to maintain participants’ privacy and to ensure the confidentiality of the collected data.

### Data analysis plan

The data were coded and analysed using the Statistical Package for the Social Sciences (SPSS®), version 22 (SPSS Inc., IBM Corp., Armonk, NY, USA). Both descriptive and inferential statistics were applied to summarise the study results. We used chi-squared tests to assess the relationship between UI and study covariates (age, BMI, T2DM duration, HbA_1c_, T2DM complications, smoking, history of UTIs, and parity). Multivariate logistic regression analyses were then performed to adjust for confounders and to estimate the risk factors for UI using odds ratios (ORs) and 95% CIs. A *P* < 0.05 was set as a statistically significant value.

## Results

Of the 400 North West Bank (Palestine) female diabetic patients who were invited to participate in the study, 19 of them refused to be involved as they justified that they had no time for the interview or felt embarrassed to talk about UI. Therefore, the research analysis included 381 participants.

Patients’ age ranged from 30 to 83 years, with a mean (SD) of 58 (10.0) years; 62.2% of them where obese, 31% were physically active, and 11.3% were smokers. About 70% of the female T2DM patients had ≥5 normal deliveries and almost half of them had a history of abdominal and/or genitourinary surgeries. For DM status, 47.9% of the patients had had DM for ≥10 years and 66.4% were on non-insulin therapy, including oral hypoglycaemic agents and life-style modifications. Of the patients, 69.9% had a history of recurrent UTI and 59.0% have chronic T2DM complications including: retinopathy, nephropathy, neuropathy, and diabetic foot. Table 1 presents in detail the demographics of the patients.

**Table 1.** Background and clinical characteristics of female diabetic patients (*n* = 381).

Variable	*N* (%)
Age (years) <50 50–59.9 60–69.9 ≥70	69 (18.1) 141 (37.0) 117 (30.7) 54 (14.2)
BMI (kg/m^2^) <18.5 18.5–24.9 25–29.9 30–34.9 ≥35	1 (0.3) 25 (6.7) 111 (29.7) 117 (30.7) 120 (31.5)
Smoking No Yes	338 (88.7) 43 (11.3)
Regular sport Yes No	120 (31.5) 259 (68.0)
Parity (*n*) <5 ≥5	114 (29.9) 256 (69.6)
History of abdominal/genitourinary surgeries No Yes	187 (49.3) 192 (50.7)
HbA_1c_ (%) <7 ≥7	110 (31.6) 238 (68.4)
Duration of DM (years) <10 ≥10	196 (52.1) 180 (47.9)
Type of treatment Non-insulin Insulin	253 (66.4) 128 (33.6)
Complications of DM* No Yes	154 (40.1) 218 (59.0)
History of recurrent UTI No Yes	114 (30.1) 265 (69.9)

*Retinopathy, nephropathy, neuropathy, diabetic foot.

The prevalence of UI amongst female diabetic patients regardless of the type was 43.2% (95% CI 37.9–47.8%), and 55.6% of them had it on a daily and weekly basis. Amongst the patients with UI, 133 (80.6%) and 128 (77.6%) were found to have UUI and SUI, respectively. Mixed UI was reported in103 (62.4%) of the patients. Almost two-thirds (65.4%) of the patients had UUI, and 43.2% of those with SUI had it on daily or weekly basis. Amongst patients with UI, 21% had leakage in the form of drops. Table 2 summaries the prevalence and frequencies of the different types of UI.

**Table 2.** Prevalence of UI in female diabetic patients: any UI, SUI and UUI.

Type of UI	*N* (%)
**Any UI**	
Yes	165 (43.2)
No	216 (56.8)
Frequency (*n* = 165)	
Less than once a month	41 (25.3)
Few times a month	31 (19.1)
Weekly	36 (22.2)
Daily	54 (33.4)
**SUI** (*n* = 165)	
Yes	128 (77.6)
No	37 (22.9)
Frequency (*n* = 128)	
Less than once a month	38 (30.4)
Few times a month	33 (26.4)
Weekly	21 (16.8)
Daily	33 (26.4)
**UUI** (*n* = 165)	
Yes	133 (80.6)
No	32(19.4)
Frequency (*n* = 133)	
Less than once a month	19 (14.3)
Few times a month	27 (20.3)
Weekly	31 (23.3)
Daily	56 (42.1)
**Mixed UI** (*n* = 165)	
Yes	103(62.4)
No	52(37.6)

Patients were asked about the degree to which UI bothered them and affected their daily life. About 40% of them reported that they were extremely bothered by the condition and 35.2% stated that their daily routine life was greatly affected (Table 3).

**Table 3.** Perceived impact of UI on female diabetic patients and their daily activities (*n* = 165).

Variable	*N* (%)
**Bothering the patients**	
Not at all	40 (24.7)
Little or somehow	58 (35.8)
Highly bothered	63 (38.9)
**Affect their daily routine life**	
Not at all	49 (29.7)
Little or somehow	57 (34.6)
Highly affected	58 (35.2)

The UI status was studied in relation to patients’ background and clinical characteristics and showed a significant relationship between UI and age (*P* < 0.001), BMI (*P* = 0.04), parity (*P* = 0.04), and history of recurrent UTI (*P* < 0.001). Whereas there was no evidence of a significant relationship between UI and T2DM duration, HbA_1c_, type of treatment, T2DM complications, and previous abdominal/genitourinary surgeries (Table 4).

**Table 4.** Patients’ characteristics in relation to UI amongst female diabetic patients (*n* = 381).

Variables	UI		*P*	OR (95% CI)
	Yes, *n* (%)	No, *n* (%)		
Age (years)				
<65	116 (43.4)	151 (56.6)	0.873	0.98 (0.63–1.5)
≥65	49 (43.0)	65 (57.0)		
BMI (kg/m^2^)				
<30	50 (36.5)	87 (63.5)	0.042	1.5 (1.08–2.4)
≥30	111 (46.8)	126 (53.2)		
Parity (*n*)				
<5	58 (35.1)	96 (45.5)	0.040	1.6 (1.12–2.5)
≥5	107 (64.8)	115 (54.5)		
Previous abdominal/ genitourinary surgeries				
Yes	90 (46.9)	102 (53.1)	0.184	1.3 (0.90–2.0)
No	75 (40.1)	112 (59.9)		
Regular sport				
Yes	41 (24.8)	79 (37)	0.152	0.62 (0.24–1.3)
No	124 (75.2)	134 (63)		
Smoking				
Yes	33 (76.6)	10 (23.3)	0.642	084 (0.4–1.87)
No	246 (73.4)	89 (26.6)		
HbA_1c_ (%)				
<7	44 (40.0)	64 (60.0)	0.517	1.2 (0.73–1.9)
≥7	104 (43.7)	134 (56.3)		
Duration of DM (years)				
<10	77 (39.3)	119 (60.7)	0.077	1.5 (0.98–2.2)
≥10	87 (48.3)	93 (51.7)		
Type of treatment				
Insulin	62 (48.4)	66 (51.6)	0.151	1.4 (0.92–20.1)
Non-insulin	103 (40.7)	150 (59.3)		
History of recurrent UTI				
Yes	134 (51.1)	128 (48.9)	<0.001	3.1 (1.9–5.1)
No	28 (25.0)	84 (75.0)		
Complication of DM				
Yes	105 (46.3)	122 (53.7)	0.158	1.3 (0.90–2.4)
No	60 (39.0)	94 (61.0)		

After using multiple logistic regression to adjust for potential confounding factors (age, BMI, T2DM duration, history of UTIs, and parity), history of recurrent UTI (adjusted odds ratio [OR] 3.0, 95% CI 1.9–4.9; *P* < 0.001) and parity (adjusted OR 1.7, 95% CI 1.1–2.7; *P* = 0.04) remained significant and predicted the occurrence of UI amongst diabetic women (Table 5).

**Table 5.** Adjusted association of UI amongst female diabetic patients (*n* = 381).

Variables	SE of OR	Adjusted OR (95% CI)	*P*
Age (years)			
<65	0.254	0.934 (0.60–1.6)	0.93
≥65			
BMI (kg/m^2^)			
<30	0.232	1.5 (0.94–2.3)	0.98
≥30			
Parity (*n*)			
<5	0.249	1.7 (1.1–2.7)	0.042
≥5			
Duration of DM (years)			
<10	0.228	1.4 (0.90–2.1)	0.206
≥10			
History of recurrent UTI			
Yes	0.259	3.0 (1.9–4.9)	<0.001
No			

**SE**, standard error.

## Discussion

Recent research has confirmed the significant relationship between DM and UI. Evidence has shown that diabetic women are at 50–200% greater risk of developing UI compared to women with normal glucose levels [12]. In the present study, the prevalence of UI amongst Palestinian female diabetic patients regardless of the type was 43.2%. This is consistent with the results of other studies in different developing and developed countries, e.g. Jordan (44%) [3] and Turkey (41%) [14]. Whilst, other countries have a higher prevalence of UI, e.g. the United Arab Emirates (63%) [4] and Kuwait (95.2%) [15].

The variation in the literature in the prevalence of UI could be attributed to different definitions of UI, different variables and inclusion criteria applied in each study, and to different settings and tools used for data collection. The high prevalence of UI reported in our present sample may support the hypothesised effect of DM on bladder function as a consequence of a hyperglycaemic effect and microvascular complications [10]. Such a finding could carry important implications for primary healthcare physicians working with Palestinian women. According to the current American Diabetes Association *Standards of Medical Care in Diabetes –* 2012 [16], assessment of diabetes-related complications, including bladder dysfunction, should be carried out routinely as an essential component of the comprehensive diabetes evaluation. In the present study, of those women who reported UI, most of them (55.6%) had it on a weekly and daily basis.

UUI is more common amongst diabetic women than non-diabetics. In our present study, 133 (80.6%) of the women with UI had UUI and 128 (77.6%) had SUI. The predominance of UUI amongst our patients is supported by other studies [17], which have proposed that diabetic microvascular complications, specifically neuropathy, reduce the continence function of the bladder.

The emotional impact of UI, including social and recreational isolation from fear and anxiety of being incontinent in public, was reported by Sinclair et al. [18]. The women in that study were asked about the social and emotional impact of UI on their daily life; 58 (35.2%) of the incontinent women reported that their daily routine life was greatly affected by UI. Additionally, 63 (38.9%) of them reported that they were highly bothered with their condition. Findings on the impact of UI on QoL are inconsistent between different Arab countries. A study in Qatar showed that UI had a moderate-to-severe impact on QoL in 42.6% and a severe impact in 49.8% of the women [19]. In Kuwait, 52% of women reported that they were either not affected ‘at all’ or only had ‘little’ impact on their lives [15]. Such findings could be attributed to the fact that the rating of QoL may be different between people depending on socioeconomic class, education, co-morbidities, political stability, and other factors. This is supported by findings of some studies that UI is often not reported and undertreated [20], which could carry important implications for primary healthcare physicians; they should be alert for UI and evaluate it in their patients.

There is great incongruence in the literature regarding the different contributing factors to the risk of UI amongst female diabetic patients. In the present multivariate analysis, history of recurrent UTI (*P* < 0.001) and parity (*P* = 0.04) were found to have a strong association with UI. Whereas there was no relationship between age, BMI, T2DM duration, type of treatment, and complications of T2DM, with UI.

We did not find a significant relationship between age and UI, contrary to findings from previous studies [3,7,14]. Age is known to be associated with decreased sensation, detrusor muscle mass, elasticity and capacity of the bladder, which cause UI manifestations [21].

In the present cohort, a history of recurrent UTIs was found to be the main risk factor and predictor of UI amongst the diabetic women; a history of recurrent UTI tripled the risk of UI. Nazarko [21] (2010) stated that diabetes doubled the risk of UTIs, which makes the ‘irritable bladder more irritable’, therefore causing the bladder to become incontinent. This emphasises the need for early diagnosis and management of UTIs to prevent the development of recurrent UTIs. Furthermore, health education in diabetic women to increase awareness about signs and symptoms, reinforcing personal hygiene, and drinking more fluids, is needed.

In the univariate analysis, the results indicated that the risk of UI in diabetic women increased with greater BMI; women with a BMI ≥30 kg/m^2^ were 1.5-times more likely to develop UI. However, it did not remain significant in multivariate analysis. This could be explained by the fact that majority of our present diabetic women were obese. Abdominal and genitourinary surgeries are a known risk factor for UI. Our present study found that diabetic women with a history of surgeries were 1.3-times more likely to have UI compared to women without a history of surgeries (OR 1.3); however, this difference was not statistically significant.

The type of T2DM management, insulin vs non-insulin therapy, was not found to be significantly associated with UI, which is consistent with findings from prior research [7,14]. Such results support the suggestion that diabetes dramatically changes the function of the bladder irrespective of the type of diabetes management.

However, our present study has some limitations that should be taken into consideration while interpreting its results. Using a cross-sectional study design to conduct the study limits the causal inferences that can be made; thus, future studies should use prospective longitudinal designs to establish causal relationships. Secondly, the validity of self-reported data has been called into question by some authors. Additionally, waist circumference could be a better measure of abdominal obesity, which in turn could have an impact on bladder function.

In conclusion, UI is a prevalent health problem, especially amongst diabetic patients. In the present study, 43.2% of the Palestinian female diabetic patients had UI. Furthermore, UI is considered bothersome and has a greater effect on daily activities in this population. The number of children delivered (parity) and a history of recurrent UTI were found to increase the risk of UI. Primary healthcare physicians should be alert for UI amongst their diabetic women, which is often unrecognised and therefore undertreated. In particular, those with a history of recurrent UTI should be given more attention. Diabetic women should be counselled and offered interventions to prevent or improve UI; such as, weight loss, stopping hormone therapy, and therapies that improve or prevent microvascular disease, including glycaemic control and blood pressure control. Future studies are needed to further explore risk factors for UI.
